# Effects of *Bifidobacterium animalis* Subsp. *lactis* (BPL1) Supplementation in Children and Adolescents with Prader–Willi Syndrome: A Randomized Crossover Trial

**DOI:** 10.3390/nu12103123

**Published:** 2020-10-13

**Authors:** Montse Amat-Bou, Sonika Garcia-Ribera, Eric Climent, Irene Piquer-Garcia, Raquel Corripio, David Sanchez-Infantes, Laia Villalta, Maria Elias, Josep C. Jiménez-Chillarón, Empar Chenoll, Daniel Ramón, Lourdes Ibañez, Marta Ramon-Krauel, Carles Lerin

**Affiliations:** 1 Institut de Recerca Sant Joan de Déu, 08950 Barcelona, Spain; moamat@sjdhospitalbarcelona.org (M.A.-B.); sgarciaribera@vhebron.net (S.G.-R.); lvillalta@sjdhospitalbarcelona.org (L.V.); meliasa@sjdhospitalbarcelona.org (M.E.); jjimenezc@fsjd.org (J.C.J.-C.); Libanez@sjdhospitalbarcelona.org (L.I.); mramonk@sjdhospitalbarcelona.org (M.R.-K.); 2 Endocrinology Department, Hospital Sant Joan de Déu, 08950 Barcelona, Spain; 3 Archer Daniels Midland Co-Biopolis, 46980 Valencia, Spain; Eric.Climent@adm.com (E.C.); Maria.Chenoll@adm.com (E.C.); Daniel.RamonVidal@adm.com (D.R.); 4 Department of Endocrinology and Nutrition, Germans Trias i Pujol Research Institute, 08916 Barcelona, Spain; irenepiquergarcia@gmail.com (I.P.-G.); dsanchez@igtp.cat (D.S.-I.); 5 Service of Pediatric Endocrinology, Parc Taulí Hospital Universitari, Institut d’Investigació i Innovació Parc Taulí I3PT, Universitat Autònoma de Barcelona, 08208 Sabadell, Spain; rcorripio@tauli.cat; 6 CIBEROBN, Instituto de Salud Carlos III, 28029 Madrid, Spain; 7 Child and Adolescent Psychiatry and Psychology Department, Hospital Sant Joan de Déu, 08950 Barcelona, Spain; 8 CIBERDEM, Instituto de Salud Carlos III, 28029 Madrid, Spain

**Keywords:** obesity, Prader–Willi syndrome, insulin sensitivity, hyperphagia, gut microbiota, probiotic supplementation, mental health

## Abstract

Prader–Willi syndrome (PWS) is a rare genetic disorder characterized by a wide range of clinical manifestations, including obesity, hyperphagia, and behavioral problems. *Bifidobacterium animalis* subsp. *lactis* strain BPL1 has been shown to improve central adiposity in adults with simple obesity. To evaluate BPL1′s effects in children with PWS, we performed a randomized crossover trial among 39 patients (mean age 10.4 years). Participants were randomized to placebo–BPL1 (*n* = 19) or BPL1–placebo (*n* = 20) sequences and underwent a 12-week period with placebo/BPL1 treatments, a 12-week washout period, and a 12-week period with the crossover treatment. Thirty-five subjects completed the study. The main outcome was changes in adiposity, measured by dual-energy X-ray absorptiometry. Secondary outcomes included lipid and glucose metabolism, hyperphagia, and mental health symptoms. Generalized linear modeling was applied to assess differences between treatments. While BPL1 did not modify total fat mass compared to placebo, BPL1 decreased abdominal adiposity in a subgroup of patients older than 4.5 years (*n* = 28). BPL1 improved fasting insulin concentration and insulin sensitivity. Furthermore, we observed modest improvements in some mental health symptoms. A follow-up trial with a longer treatment period is warranted to determine whether BPL1 supplementation can provide a long-term therapeutic approach for children with PWS (ClinicalTrials.gov NCT03548480).

## 1. Introduction

Prader–Willi syndrome (PWS) is a rare genetic disorder characterized by a wide range of clinical manifestations, including altered body composition, hyperphagia, and severe behavioral problems [1]. It is considered the most common cause of genetic obesity due to a combination of low energy expenditure and high energy intake [2,3,4]. PWS is caused by the lack of expression of genes from the paternal chromosome 15q11–q13 region [1,2]. The most frequent mechanisms are deletions (65%–75%) and maternal disomy (20%–30%), with imprinting defects and translocations being the least frequent [1,2]. The natural history of this disease is characterized by a complex progression through distinct nutritional phases since birth [5]. Patients present hypotonia, feeding difficulties, and failure to thrive during the first months of life, and then transition toward increased appetite, interest in food, and excessive adiposity at approximately 4.5 years of age. Later on, they typically develop hyperphagia, morbid obesity if uncontrolled, and mental health problems, including anxiety and social problems [5,6], with patients with maternal disomy at higher risk for more severe problems [7]. While current therapies have improved health outcomes during the last decade [8], obesity and mental health problems continue to be an enormous burden for patients and families.

The gut microbiome is recognized as an important player in the development not only of obesity and metabolic disease [9,10] but also of mental health disorders including depression, anxiety, and social behavior [11,12,13]. Several *Bifidobacterium animalis* subsp. *lactis* strains have been shown to improve cardiometabolic risk factors in adult subjects with obesity by decreasing central adiposity and insulin resistance [14,15,16]. Specifically, strain CECT 8145 (BPL1) was originally identified as a potent probiotic, decreasing fat deposition in a high-throughput screening in *Caenorhabditis elegans* [17]. In this organism, BPL1 targets both energy homeostasis and tryptophan metabolism [17], an important regulator of central nervous system processes including satiety, anxiety, and depression [13,18]. Only a small number of studies have examined the gut microbiome in PWS [19,20,21,22]. Zhang et al. studied the effects of a hospitalized hypocaloric dietary intervention on 17 children and adolescents with PWS and showed that diet-induced changes in the gut microbiome directly contributed to improved adiposity and metabolic health [19]. Olsson et al. studied the gut microbiome in 17 adult subjects with PWS and a matched group with non-genetic obesity, concluding that gut bacteria play an active role in insulin sensitivity [20]. We recently reported the gut microbiota associated with obesity in children with PWS, finding similar alterations to those found in simple obesity [21]. Finally, Peng et al. described several differences in intestinal bacterial genera and fungal communities in children with PWS compared to those in controls [22].

The main aim of the present study was to determine the potential benefits of probiotic BPL1 on cardiometabolic risk factors in subjects with PWS. To this aim, we conducted a randomized controlled crossover study to compare the effects of BPL1 strain and placebo supplementation in children and adolescents with PWS. The main outcome was change in adiposity, and secondary outcomes included lipid and glucose homeostasis, hyperphagia, and mental health symptoms.

## 2. Materials and Methods

### 2.1. Study Design

Given the characteristics of this disease, specifically the very low prevalence and the heterogeneity of clinical and demographic parameters (wide age range, obesity status, and genetic mechanisms), a randomized crossover design with a 1:1 allocation ratio was chosen for this study. The study consisted in a first treatment period of 12 weeks when participants received probiotic or placebo (Period 1), a 12-week washout period, and finally a second treatment period of 12 weeks when participants received the crossover treatment (Period 2). All parents gave their informed consent, and children older than 12 years gave their informed assent for inclusion before they participated in the study. The study was conducted in accordance with the Declaration of Helsinki, and the protocol was approved by the Ethics Committee of Hospital Sant Joan de Déu (Project identification code: PIC-73-17). The study was registered at ClinicalTrials.gov (ID number: NCT03548480, https://clinicaltrials.gov/ct2/show/NCT03548480).

### 2.2. Study Participants

Participants were recruited between January 2018 and June 2018 from the Hospital Sant Joan de Déu (Barcelona, Spain) and the Parc Taulí Hospital Universitari (Sabadell, Spain), which are the two reference centers in Catalonia for patients with PWS. All visits and tests were performed at Hospital Sant Joan de Déu. Inclusion criteria were genetically confirmed PWS diagnosis, age between 2 to 19 years, and being on a stable diet and medication regimen for at least two months before enrollment, with special attention to growth hormone (GH) therapy and metformin use. Exclusion criteria included current enrollment in or discontinuation within the last 30 days from a clinical trial, presence of other medical problems that would preclude study participation, history of bariatric surgery, and unsuitability for inclusion in the opinion of the investigator. Sample size was determined considering the low prevalence of PWS (1:15,000 births, with an average of approximately 75,000 births per year in Catalonia) and based on the existing number of patients at both hospitals. Considering this, 30 participants would provide a power of ≥80% to detect a statistically significant difference (two-sided, significance level of 5%) of at least 1.1 units in the change of % total body fat mass (primary outcome) in a paired comparison between placebo and BPL1 periods. This was based on an SD of 2.1 in the change of % total body fat mass over approximately one year from previous observations at our clinical settings.

### 2.3. Intervention

Probiotic and placebo were administered daily as an oral supplement. They were provided as opaque capsules containing 100 mg of *Bifidobacterium animalis* subsp. *lactis* (BPL1, CECT8145, 10^10^ colony forming units) with 200 mg of maltodextrin (used as a carrier during the lyophilization process), or 300 mg of maltodextrin, respectively. Both BPL1 and placebo capsules were obtained from Archer Daniels Midland Co-Biópolis (Valencia, Spain). Treatment was provided at the baseline visit of each period, and parents were instructed to store the capsules in a refrigerator (4 °C). Compliance was assessed by counting the remaining capsules at the end of each period, and 96% of patients had a compliance over 80% in both periods (83% of participants had higher than 90%). Patients were advised to not make dietary changes or take any nutritional supplements or other probiotics throughout the duration of the study. Compliance with the recommendations was assessed at each visit with the dietitian; none of the participants made any significant changes to their diet during the duration of the study or received any probiotic other than the one provided by the study team. Potential adverse effects were monitored throughout the study. Patients and families were instructed to reach out to the study team if they noticed any symptoms or illness that could be related or not to the treatment. Additionally, they were proactively questioned at each study visit about potential adverse events or problems, including treatment tolerance, abdominal pain, and diarrhea. We did not consider any adverse event from any previously scheduled surgery or hospital admission to be related to the study.

### 2.4. Randomization

Study participants were randomly assigned to a treatment sequence (AB/BA, where A is placebo and B is BPL1) by an investigator external to the study according to a computer-generated allocation schedule, by block randomization (2 × 2), and stratifying by sex and age (8-year-old). Staff involved in measuring and analyzing outcomes were blinded to the intervention allocation.

### 2.5. Outcomes

All outcomes were measured at the four time points of the study. The main outcome was change in adiposity assessed by dual-density X-ray absorptiometry (Lunar Prodigy^®^ DXA instrument, Lunar Corp., Madison, WI, USA). Other variables included height and body weight, as well as blood pressure. Age- and sex-adjusted body mass index standard deviation scores (BMI-SDS) were calculated using WHO Anthro and AnthroPlus software (World Health Organization, Geneva, Switzerland) [23]. Fasting blood samples were obtained at all visits to measure triglyceride, total cholesterol, low-density lipoprotein (LDL)- and high-density lipoprotein (HDL)-cholesterol levels, as well as glucose, insulin, and hemoglobin A1c (HbA1c) concentrations. Homeostatic model assessment of insulin resistance (HOMA-IR) was calculated as previously described [24]. We also collected parental-reported 4-day food records that were analyzed with the DIAL software [25] to assess daily energy intake as previously reported [21]. Hyperphagia was assessed with the Hyperphagia Questionnaire for Clinical Trials (HQ-CT, validated Spanish version), a nine-question questionnaire (score 0–36) developed for patients with PWS. To evaluate mental health problems, we used the Childhood Behavior Check List (CBCL), which is a component of the Achenbach System of Empirically Based Assessment (ASEBA). Specifically, we used the validated Spanish version of the parental-reported form for subjects between 6 and 18 years old. This test measures emotional and behavioral problems using over 100 items, grouped into Internalizing, Externalizing, and Total scales. It consists of 8 subscales (Anxious/depressed, Withdrawn/depressed, Somatic complaints, Social problems, Thought problems, Attention problems, Rule-breaking behavior, and Aggressive behavior). It has satisfactory reliability and validity in general, clinical, and intellectually disabled populations, including patients with PWS [26].

### 2.6. Subgroup Analyses

Given the participants’ wide age range and its potential implication in the outcome variables, we performed subgroup analyses stratifying by age under different premises. First, young children with PWS (phase I and IIa) show slow growth and failure to thrive, as well as a wide variation in adiposity [5]. At 4.5 years of age (entering phase IIb of the disease) they start showing increased appetite and excessive weight gain and adiposity [5]. Therefore, we performed a secondary analysis of the effects of BPL1 on adiposity in the subgroup of participants older than 4.5 year of age (*n* = 28). Furthermore, behavioral issues are highly age dependent, being more exacerbated in older children. Consequently, we used the CBCL suitable for ages between 6 and 18 years to assess the effects of BPL1 on behavior in the subgroup of participants older than 6 years (*n* = 20 completed the CBCL at the four time points). Finally, given that PWS-associated mental health problems (e.g., autism spectrum symptoms) occur more frequently in maternal disomies than in deletions [6,27], we also performed a subgroup analysis of CBCL scores stratifying by genotype.

### 2.7. Gut Microbiota 

Stool samples at the four time points of the study were available from 33 subjects. DNA from these samples was isolated with the QIAamp PowerFecal DNA Kit (Qiagen, Hilden, Germany) as previously described [28] with minor modifications to avoid bias in DNA purification toward misrepresentation of Gram-positive bacteria. A MiSeq Illumina Platform (Illumina, Sant Diego, CA, USA) was used following Illumina recommendations to sequence the hypervariable region V3–V4 of the bacterial 16S gene using key-tagged eubacterial primers [29], obtaining an average number of 85,000 reads per sample. “bbmap” tools v37.50 [30] were applied to overlap R1 and R2 reads and obtain a single FASTQ file for each sample. Quality control of the sequences was performed in different steps. First, 16S primers were trimmed from the sequences using Cutadapt v2.6 [31]. Next, quality filtering was performed with “bbmap” tools v37.50, and all sequences with an average quality score under 20 in the Phred scale were removed. In this step, sequences shorter than 200 nucleotides were discarded too. The resulting sequences were inspected for PCR chimera constructs that may occur during the different experimental process. Those chimeric sequences were identified and removed from further analysis using cd-hit 4.8.1 [32]. Resulting high-quality FASTA files were used to create an operational taxonomic unit (OTU) table with cd-hit 4.8.1 software. A total amount of 224.713 high-fidelity OTUs were clustered, with 99% identity within them. Those OTUs were compared against the National Center for Biotechnology Information (NCBI) 16S rRNA database using BLASTn version 2.10. The resulting XML files were processed using a python script developed by ADM Lifesequencing (Valencia, Spain) to annotate each sequence at different phylogenetic levels. In order to reduce noise in the analysis, OTUs not present in at least 50% of the samples of a given group were removed. The 2274 remaining OTUs contained 67% of the original sequences. 

### 2.8. Statistical Analysis

Unless otherwise stated, data are shown as mean and standard deviation (SD) or n and percentage (%) for continuous and categorical variables, respectively. Comparisons for baseline variables between sequence allocation groups (AB and BA) were performed using unpaired Student’s *t*-test. Comparison of categorical variables among sequence groups was assessed using chi-squared test. Shapiro–Wilk’s test was used to assess normality before further analysis. Microbiota data were analyzed comparing log2 fold changes of count number between placebo and BPL1 treatment periods with paired Wilcoxon signed-rank test. To estimate treatment effects on physiological and mental health variables, we calculated the change in outcome values for each treatment period (P1 and P2), and then the difference between periods (P2–P1). Multivariate generalized linear modeling was then performed with treatment sequence (AB/BA) as independent variable and the difference between periods of outcomes (P2–P1) as dependent variable. Where indicated, co-variates such as age, sex, genotype, and baseline adiposity were included to adjust the statistical models. Results are shown as effect size (B) with 95% confidence interval (CI). A *p* < 0.05 was considered significant. Analyses were performed in JMP^®^ v14.3 (SAS, Cary, NC, USA). 

## 3. Results

### 3.1. Study Participants

**Figure 1:**
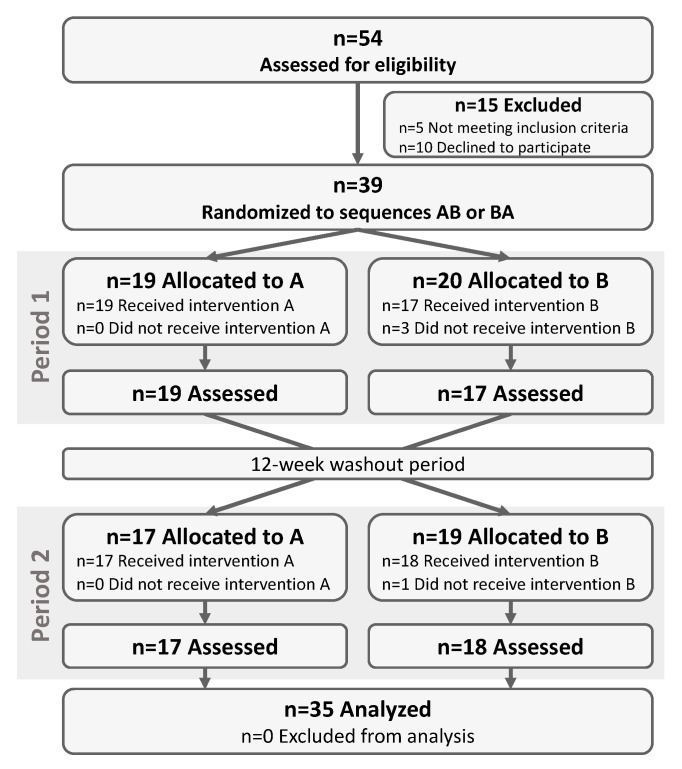
Flow chart of the study participants. The flow chart of participants throughout the study is shown. A corresponds to placebo treatment and B to *Bifidobacterium animalis* subsp. *lactis* strain BPL1 probiotic treatment. AB, treatment sequence placebo-BPL1; BA, treatment sequence BPL1-placebo.

The flow diagram for the clinical study is shown in Figure 1. Among the 54 patients that were assessed for eligibility, 39 met the inclusion criteria and accepted to participate. These subjects were randomized to sequence AB (*n* = 19, placebo–BPL1) or BA (*n* = 20, BPL1–placebo). One subject from sequence BA voluntarily dropped out of the study during the first visit before starting any treatment. Two other subjects randomized in sequence BA voluntarily dropped out of the study during Period 1 after a viral gastroenteritis that also affected other members of the family and after an episode of exacerbated behavioral problems that persisted after treatment discontinuation, respectively. Finally, another subject from sequence AB reported loss of the treatment capsules at the beginning of Period 2 and decided to drop out of the study. The remaining 35 participants completed the study and were included in the analysis (Figure 1). 

The baseline characteristics of participants are described in Table 1. The mean age of these subjects was 10.4 years, and they showed an average body fat mass content of 40.8%. Lipid profile, glucose, and HbA1c levels were within the normal range. Fasting insulin concentration was on the upper end of the normal range in our laboratory (<19.5 mU/L), resulting in average HOMA-IR values over 2, indicating the presence of some degree of insulin resistance. There were no differences between sequence groups at baseline, except for fasting glucose concentration, which was slightly lower in AB compared to that in the BA group (Table 1).

### 3.2. Effects of BPL1 Probiotic Supplementation on Gut Microbiota Composition

**Figure 2:**
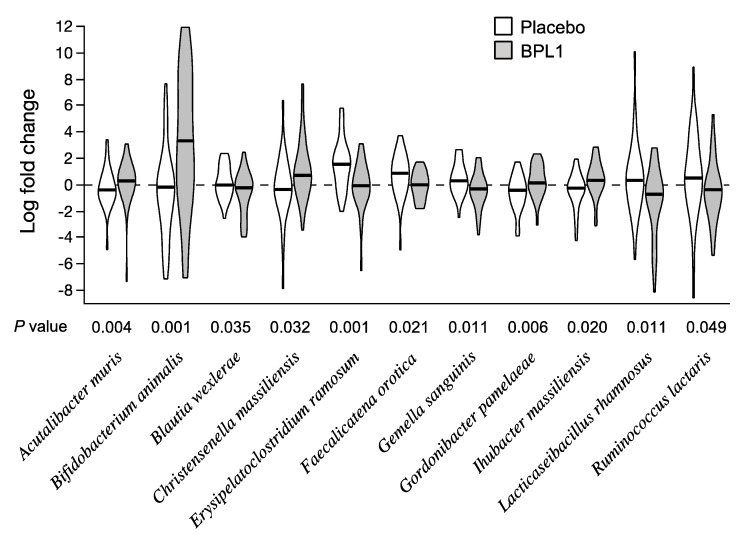
Changes in abundance of species during placebo and BPL1 treatment periods. Violin charts show log2 fold changes during placebo (white) and probiotic (grey) periods. Horizontal bars correspond to median values of log2 fold change. *p* values are from paired Wilcoxon signed-rank test between placebo and probiotic groups.

Microbiota composition was not substantially modified by probiotic treatment, and no significant differences were obtained at genus level. Therefore, results are shown at species level. Figure 2 shows the species with differential change during the placebo and BPL1 treatment periods (paired Wilcoxon signed-rank *p* < 0.05). *Bifidobacterium animalis* abundance showed the greatest difference between probiotic and placebo treatment periods (Figure 2). Other species significantly altered in the treatment comparison included increases in *Acutalibacter muris*, *Christensenella massiliensis*, *Gordonibacter pamelaeae*, and *Ihubacter massiliensis*, and decreases in *Blautia wexlerae*, *Erysipelatoclostridium ramosum*, *Faecalicatena orotica*, *Gemella sanguinis*, *Lacticaseibacillus rhamnosus* (formerly *Lactobacillus rhamnosus*), and *Ruminococcus lactaris* (Figure 2). Log2 fold changes for placebo and BPL1 treatment periods and *p* values from paired tests for all species are shown in Table S1.

### 3.3. Effects of BPL1 Probiotic Supplementation on Adiposity

No differences in total body fat mass were observed between treatment assignments, except for a trend toward decreased abdominal fat mass during the BPL1 treatment period compared to that for placebo (Table 2). However, we observed a significant decrease in abdominal fat mass after BPL1 supplementation compared to placebo when performing a subgroup analysis including only subjects older than 4.5 years (Table 2). Baseline characteristics of this subgroup are shown in Table S2. Absolute values for total and abdominal fat mass at each time point are shown in Table S3. No period-dependent effects were observed for these variables (Table S4).

### 3.4. Effects of BPL1 Probiotic Supplementation on Hyperphagia, Lipid Profile, and Glucose Homeostasis 

BPL1 had no effects on hyperphagia or energy intake compared to placebo (Table 3). Furthermore, we observed no differences in blood pressure, lipid profile, glucose, and HbA1c levels (Table 3). Notably, BPL1 supplementation significantly decreased fasting insulin concentration and HOMA-IR compared to placebo (Table 3). Values of insulin and HOMA-IR for each time point are shown in Table S3. No period-dependent effects were observed (Table S4).

One of the participants displayed a divergent pattern of fasting insulin concentration at the different time points, showing as a clear outlier when the difference between periods was analyzed (Figure S1A,B). This participant was an 11-year-old female with baseline BMI-SDS −0.23, born at 28 weeks of gestation (very preterm birth). Given that prematurity was not defined as an exclusion criterion, this participant was originally included in the study. Excluding this subject resulted in a more robust treatment effect on fasting insulin concentration (B = −5.65, *p* < 0.001) and HOMA-IR (B = −1.34, *p* = 0.001) (Figure S1C).

### 3.5. Effects of BPL1 Probiotic Supplementation on Mental Health Problems

Mental health problems were evaluated in the subgroup of participants older than 6 years of age, using the parent-report form of the CBCL for subjects between 6 and 18 years of age. The checklist was completed at the four times by 20 of the study subjects. The baseline CBCL subscale scores for these subjects are shown in Table S5. Although there were no statistically significant changes in overall psychopathological levels or in the internalizing or externalizing subdomains, we observed a moderate effect of BPL1 on some symptom dimensions (Table 4). Specifically, BPL1 induced modest but significant improvements (decrease in test score) in withdrawn/depression symptoms, and a trend toward improvement in attention problems compared to placebo. The rest of the symptom dimensions evaluated did not reach statistical significance, although several showed numerical decreases (Table 4). 

Given that the risk for severe mental health problems is higher in subjects with maternal disomy than in those with deletions [7], we performed an exploratory analysis to compare the effects of BPL1 supplementation between both genotypes. We observed no differences at baseline between subjects with deletions (*n* = 11) and disomies (*n* = 9) (Table S5). Strikingly, improvements in mental health symptoms largely happened in subjects with maternal disomy, while they were only marginal in subjects with deletions (Figure S2). Significant differences between genotypes were observed in withdrawn/depression symptoms and attention problems (*p* = 0.036 and *p* = 0.026, respectively) (Figure S2). 

### 3.6. Adverse Events

Probiotic and placebo were well tolerated, and no serious adverse events were observed during the study. None of the participants reported abdominal pain. Diarrhea was reported in 6 patients (4 during probiotic and 2 during placebo period). All episodes of diarrhea resolved in a few days and were attributed to viral gastroenteritis. One participant reported exacerbated behavioral problems during probiotic treatment, which persisted after treatment discontinuation. No other adverse events were reported.

## 4. Discussion

Increasing evidence over the last years shows that probiotic supplementation can provide a therapeutic strategy for obesity and metabolic disease [14,15,16,33]. Specifically, the beneficial metabolic effects of *B. animalis* subsp. *lactis* in humans are well known, with three different strains (including the BPL1 strain) improving central adiposity in adults with overweight or mild obesity [14,15,16,34]. Our study with the BPL1 strain in subjects with PWS demonstrates that this probiotic exerts beneficial effects even in the context of genetic obesity. Obesity and its co-morbidities, including type 2 diabetes and cardiovascular disease, are a major cause of premature mortality in patients with PWS. Therefore, decreasing central adiposity and improving insulin sensitivity can have a great impact on their long-term health outcomes and change the prognosis of the disorder.

Data from our study show improvements in abdominal adiposity after supplementing with probiotic BPL1 for 12 weeks compared to placebo. Notably, this effect was more evident when analyzing participants older than 4.5 years of age. It is not until approximately this age when children start showing increased interest in food and gaining excessive body weight and adiposity [5]. Our results also show that BPL1 supplementation improved insulin sensitivity. Studies in rodent models had already shown beneficial effects of different strains of *B. animalis* subsp. *lactis* on glucose homeostasis in diet-induced obesity [35,36,37]. However, data in humans are unclear. A study with BPL1 showed improvements in insulin sensitivity in adult subjects with simple obesity [16], while another clinical study with the B420 strain showed no improvements [15]. However, as highlighted by the authors of the latter study, insulin concentration in their cohort at baseline was fairly normal and left little room for improvement [15].

Data from animal and human studies suggest that the improvements in metabolic health induced by *B. animalis* subps. *lactis* could be in part mediated by decreasing energy intake. Rodent models showed reduced food intake after supplementation with BPL1 in parallel to metabolic benefits [37]. Moreover, supplementation in humans also led to decreased energy intake in one study with the B420 strain [15]. It has been suggested that these effects could be induced via the regulation of eating-related behavior, possibly through the gut–brain axis, or peptides regulating appetite and satiety, including PYY and ghrelin [18,37,38,39]. While this effect could be highly relevant in the context of PWS, hyperphagia levels and energy intake remained very stable throughout our study, suggesting that the probiotic cannot overcome the complex genetic mechanisms occurring in PWS that lead to hyperphagia. These data indicate that BPL1-induced improvements in adiposity and insulin sensitivity were independent of energy intake.

The molecular basis of BPL1 metabolic effects is not completely understood. It has been proposed that *B. animalis* subps. *lactis* acts improving the intestinal barrier function, decreasing endotoxemia and inflammation [34,35,37]. Indeed, both BPL1 and B420 strains increased *Akkermansia muciniphila* abundance in adult subjects [16,40], with well-known beneficial effects on the gut barrier function [41,42]. In contrast to another study in adult subjects [16], we did not observe major changes in gut microbiota composition in our population of children with PWS. Whether the minor effect of BPL1 on the microbiota is related to the short treatment period and relatively low number of subjects or is more specific for this disease remains unknown. In any case, *A. muciniphila* was not increased in our study, suggesting that other mechanisms are mediating BPL1′s beneficial effects in this population. Notably, a recent study has identified lipoteichoic acid as the molecule from BPL1 that mediates its metabolic effects [43]. Experiments in *Caenorhabditis elegans* showed that both the isolated molecule and the BPL1 strain act enhancing the insulin/insulin-like growth factor 1 (IGF-1) signaling pathway, which regulates metabolism, behavior, growth, and longevity in this organism [43]. The relevance of these data to PWS resides in the impaired GH/IGF-1 axis characteristic of this disease, typically with low IGF-1 levels and blunted responses to GH provocative testing [44,45]. Given that IGF-1 plays a crucial role in growth, development, and anabolic processes, it is tempting to speculate that the IGF-1 signaling pathway could be a crucial BPL1 target in these patients. Further investigations are warranted to examine the potential impact on IGF-1 signaling.

Gut bacteria are also major players in modulating important central nervous system processes, from depression to anxiety and social behavior [11,12,13]. Our results demonstrate that BPL1 induced modest improvements in some mental health problems, especially regarding withdrawn/depression symptoms and attention problems. It is interesting to note that most of the other psychopathological dimensions were also improved, although without reaching statistical significance. Anxiety, depression, and autism spectrum disorders–like symptoms (including impaired social interactions and repetitive and compulsive behavior) are common in older children with PWS and constitute a major burden for patients and families [5,6,46]. Subjects with maternal disomy are at higher risk than those with deletions for severe behavior and mental health alterations, including mood and psychotic disorders [7]. Notably, BPL1-induced improvements in behavior mostly appear in participants with disomy, while participants with deletion showed marginal changes in test scores. The potential molecular mechanisms for this differential effect are currently unknown. It is worth highlighting that treatment was for 12 weeks, and this time period might not be enough to induce robust changes in some mental health symptoms. Furthermore, the number of subjects in this analysis comparing the two genotypes is relatively low, compromising the statistical power to detect significant changes. Thus, a follow-up study with a longer treatment period and a larger cohort is warranted to confirm and further investigate the potential effects of BPL1 on behavioral aspects in the PWS context.

An intrinsic limitation when studying rare diseases and PWS is the relatively low number of patients and the high heterogeneity of patients due to the characteristics of the disease. In these regards, our cohort showed a wide age range and obesity status, as well as different genotypes. We have attempted to overcome this caveat by using a crossover design; analyzing subgroups of participants; and adjusting statistical models by different covariates, including age, sex, genotype, and baseline adiposity. Although the crossover design is crucial to minimize variability, it has the downside of potential carry over effects from the first treatment period. To minimize this effect, we introduced a 12-week washout period and checked for period effects. Another limitation is the relatively short treatment period, which might affect some of the outcome measures, especially related to behavioral aspects. In this line, the instrument used to measure these changes (CBCL) has a time frame of 24 weeks, and its capacity to detect change could be affected when using shorter time periods. 

In summary, this study shows that BPL1 probiotic supplementation improves abdominal adiposity, insulin sensitivity, and some mental health symptoms in children and adolescents with genetic obesity. Given that these beneficial effects could be crucial for the disease prognosis, a follow-up study with a longer treatment period is required to demonstrate whether supplementation with BPL1 can provide a valid long-term therapeutic strategy for patients with PWS. Finally, the implications of these results might go beyond the PWS context as data suggest that the probiotic could be equally effective in pediatric patients with simple obesity; further studies are also needed to test whether similar effects could be obtained in children and adolescents with non-genetic obesity.

## Figures and Tables

**Table 1:** Basal demographic and physiologic characteristics of participants.

	All (*n* = 35)	AB (*n* = 18)	BA (*n* = 17)	*p* Value
Sex (female)	21 (60)	12 (67)	9 (53)	0.407
Age (years)	10.4 (5.0)	10.3 (5.1)	10.6 (4.9)	0.862
Genotype (deletions)	18 (59)	12 (67)	6 (35)	0.061
Growth hormone use	34 (97)	18 (100)	16 (94)	0.225
Metformin use	8 (23)	6 (33)	2 (12)	0.122
Weight (kg)	46.9 (27.7)	47.0 (26.0)	46.7 (30.2)	0.979
Height (cm)	137.6 (26.1)	137.9 (26.6)	137.3 (26.4)	0.941
BMI-SDS	1.22 (1.45)	1.35 (1.39)	1.07 (1.55)	0.577
Body fat mass (g)	20,380 (15,952)	20,037 (14,017)	20,743 (18,213)	0.899
Body fat mass (%)	40.8 (9.7)	40.5 (9.7)	41.1 (10)	0.845
Abdominal fat mass (g)	1449 (1423)	1408 (1240)	1491 (1632)	0.867
Abdominal fat mass (%)	6.0 (1.8)	5.8 (2.1)	6.2 (1.5)	0.578
HQ-CT (Score)	6.0 (5.6)	6.1 (6)	5.9 (5.3)	0.929
Daily energy intake (kcal)	1465 (413)	1455 (463)	1475 (366)	0.891
Systolic pressure (mmHg)	108.8 (11.9)	108.4 (12.1)	109.1 (11.9)	0.870
Diastolic pressure (mmHg)	70.6 (8.7)	68.9 (8.9)	72.4 (8.3)	0.238
Triglycerides (mg/dL)	71 (24)	68 (21)	74 (26)	0.452
Total cholesterol (mg/dL)	171 (37)	176 (40)	166 (34)	0.456
LDL-cholesterol (mg/dL)	104 (32)	107 (33)	101 (31)	0.537
HDL-cholesterol (mg/dL)	56 (13)	55 (13)	56 (14)	0.778
Glucose (mg/dL)	86 (9)	82 (8)	89 (9)	**0.023**
HbA1c (%)	5.2 (0.2)	5.2 (0.2)	5.2 (0.3)	0.579
Insulin (mU/L)	11.2 (9.6)	9.9 (9.5)	12.7 (9.9)	0.508
HOMA-IR	2.49 (2.26)	2.14 (2.15)	2.89 (2.38)	0.423

Data are shown as mean and standard deviation (SD) for continuous variables and n and percentage (%) for categorical variables. Basal differences between group were assessed with Student’s *t*-test (continuous variables) or chi-square test (categorical variables). AB, treatment sequence placebo-BPL1; BA, treatment sequence BPL1-placebo. HQ-CT, Hyperphagia Questionnaire for Clinical Trials; BMI, body mass index; BMI-SDS, body mass index standard deviation score; LDL, low-density lipoprotein; HDL, high-density lipoprotein; HbA1c, hemoglobin A1c; HOMA-IR, homeostatic model assessment of insulin resistance. Bold font indicates *p* < 0.05.

**Table 2:** Treatment effects on adiposity.

	AB Group		BA Group			
	Period 1	Period 2	Period 1	Period 2	Treatment Effect	
All Subjects (*n* = 35)	Placebo	BPL1	BPL1	Placebo	B (CI 95%)	*p* Value
Body fat mass (g)	73 (1060)	58 (1166)	158 (1457)	624 (1236)	−173 (−907, 562)	0.635
Body fat mass (%)	−0.5 (1.5)	−1.3 (1.3)	−0.4 (2.2)	−0.5 (1.8)	−0.18 (−1.08, 0.73)	0.691
Abdominal fat mass (g)	−18 (177)	−28 (98)	−48 (121)	69 (140)	−62 (−133, 8)	0.082
Abdominal fat mass (%)	0.1 (0.5)	−0.1 (0.5)	−0.2 (0.6)	0.0 (0.5)	−0.23 (−0.53, 0.06)	0.121
**>4.5 Years (*n* = 28)**						
Body fat mass (g)	59 (1209)	27 (1328)	20 (1571)	489 (1312)	−156 (−1081, 768)	0.729
Body fat mass (%)	−0.5 (1.6)	−1.5 (1.3)	−1.1 (1.7)	−1.0 (1.4)	−0.30 (−1.31, 0.71)	0.548
Abdominal fat mass (g)	−26 (202)	−40 (106)	−72 (119)	72 (152)	−74 (−157, 8)	0.076
Abdominal fat mass (%)	0.0 (0.5)	−0.2 (0.4)	−0.3 (0.5)	0.1 (0.4)	**−0.33 (−0.59, −0.06)**	**0.017**

Changes between periods are shown as mean (SD). Generalized linear regression was applied to estimate treatment effects on adiposity in all subjects (*n* = 35) or older than 4.5 years (*n* = 28). The model was adjusted for sex, age, genotype, and basal % body fat mass. AB Group, treatment sequence placebo-BPL1; BA Group, treatment sequence BPL1-placebo. B, effect size; CI, confidence interval; BPL1, *Bifidobacterium animalis* Subsp. *Lactis*. Bold font indicates *p* < 0.05.

**Table 3:** Treatment effects on hyperphagia and physiologic outcomes.

	AB Group		BA Group			
	Period 1	Period 2	Period 1	Period 2	Treatment Effect	
	Placebo	BPL1	BPL1	Placebo	B (95% CI)	*p* Value
HQ-CT (Score)	−0.50 (2.12)	−1.50 (3.98)	0.65 (4.18)	−0.93 (3.38)	0.05 (−1.79, 1.89)	0.956
E.I. (kcal/day)	−2 (397)	−20 (149)	125 (199)	−31 (231)	43 (−93, 179)	0.521
SP (mmHg)	0.06 (9.55)	−1.00 (11.26)	5.07 (7.99)	2.60 (12.72)	0.95 (−4.67, 6.56)	0.730
DP (mmHg)	−0.06 (8.9)	−0.13 (12.35)	0.29 (7.89)	0.2 (8.01)	0.63 (−4.79, 6.05)	0.812
TG (mg/dL)	1.1 (28.3)	−3.4 (32.4)	−2.2 (17.1)	−7.1 (18.3)	−0.49 (−15.52, 14.53)	0.947
Total cho (mg/dL)	−5.3 (19.4)	−4.1 (28.8)	−6.1 (18.2)	−7.6 (20.2)	2.46 (−8.79, 13.71)	0.658
LDL-cho (mg/dL)	−1.2 (16.6)	3.6 (14.4)	−3.8 (16.9)	−2.1 (16.7)	1.61 (−7.25, 10.47)	0.712
HDL-cho (mg/dL)	−4.3 (10.0)	−1.8 (5.1)	−1.6 (5.8)	−4.5 (8.2)	2.31 (−2.08, 6.71)	0.290
Glucose (mg/dL)	2.4 (8.9)	−0.3 (8.9)	−1.4 (9.9)	−0.1 (10.7)	−3.21 (−8.50, 2.08)	0.225
HbA1c (%)	−0.01 (0.15)	0.06 (0.12)	−0.05 (0.12)	0.02 (0.14)	−0.01 (−0.09, 0.06)	0.690
Insulin (mU/L)	0.41 (8.34)	−2.02 (6.05)	−2.78 (9.11)	1.94 (9.26)	**−4.44 (−8.51, −0.38)**	**0.033**
HOMA-IR	0.06 (1.95)	−0.52 (1.45)	−0.70 (2.17)	0.45 (2.12)	**−1.07 (−2.04, −0.10)**	**0.031**

Changes between periods are shown as mean (SD). Generalized linear regression was applied to estimate treatment effects on hyperphagia and physiologic outcomes (*n* = 35). The model was adjusted for sex, age, genotype, and basal % body fat mass. AB Group, treatment sequence placebo-BPL1; BA Group, treatment sequence BPL1-placebo. HQ-CT, Hyperphagia Questionnaire for Clinical Trials; E.I., energy intake; SP, systolic pressure; DP, diastolic pressure; TG, triglycerides; Cho, cholesterol; LDL, low-density lipoprotein; HDL, high-density lipoprotein; HbA1c, hemoglobin A1c; HOMA-IR, homeostatic model assessment of insulin resistance; BPL1, *Bifidobacterium animalis* Subsp. *Lactis*. Bold font indicates *p* < 0.05.

**Table 4:** Treatment effects on mental health problems.

	AB Group		BA Group			
	Period 1	Period 2	Period 1	Period 2	Treatment Effect	
	Placebo	BPL1	BPL1	Placebo	B (95% CI)	*p* Value
Anxious/Depressed	−2.4 (7.3)	0.5 (1.9)	−0.3 (6.4)	−0.7 (6.3)	1.2 (−4.2, 6.7)	0.641
Withdrawn/Depressed	1.1 (5.9)	−3.0 (5.3)	−2.0 (6.0)	2.7 (3.9)	**−4.7 (−9.0, −0.3)**	**0.037**
Somatic Complaints	0.6 (3.3)	−0.1 (3.8)	−3.3 (6.2)	−0.4 (6.8)	−1.7 (−6.6, 3.2)	0.460
Social Problems	−3.3 (8.4)	0.0 (5.3)	0.6 (6.3)	1.3 (6.9)	−0.4 (−5.7, 4.8)	0.869
Thought Problems	−5.5 (7.9)	−1.3 (2.7)	−2.1 (7.4)	2.1 (5.2)	0.3 (−4.2, 4.9)	0.877
Attention Problems	−0.4 (4.2)	−2.0 (4.9)	−1.0 (3.0)	2.3 (4.9)	−2.9 (−6.5, 0.6)	0.097
Rule-Breaking Behavior	0.5 (3.5)	−2.0 (3.3)	0.7 (5.6)	0.6 (7.9)	−2.0 (−5.2, 1.2)	0.208
Aggressive Behavior	−2.0 (4.3)	−1.4 (2.5)	−0.8 (6.2)	0.3 (5.4)	0.2 (−3.7, 4.0)	0.933
Internalizing Behavior	−1.1 (6.1)	−1.9 (3.2)	−2.5 (5.9)	2.0 (5.3)	−3.0 (−8.3, 2.3)	0.249
Externalizing Behavior	−0.3 (3.6)	−2.4 (3.3)	−0.2 (4.2)	0.6 (6.1)	−1.7 (−4.4, 1.0)	0.203
Total Score	−1.4 (4.6)	−2.5 (4.8)	−1.6 (4.3)	2.3 (5)	−3.2 (−7.3, 0.9)	0.120

Changes between periods are shown as mean (SD). Generalized linear regression was applied to estimate treatment effects on the different subscales and the total score from the CBCL (*n* = 20). The model was adjusted for sex, age, and genotype. AB Group, treatment sequence placebo-BPL1; BA Group, treatment sequence BPL1-placebo. B, effect size; CI, confidence interval; BPL1, *Bifidobacterium animalis* Subsp. *Lactis*. Bold font indicates *p* < 0.05.

## Supplementary Materials

The following are available online at https://www.mdpi.com/2072-6643/12/10/3123/s1, Figure S1. Fasting insulin levels and change between periods; (A) fasting insulin levels at all visits; (B) difference of insulin levels between periods; (C) generalized linear regression was applied to assess treatment effects, Figure S2. Treatment effect on behavior by genotype, Table S1. Fold change of bacterial species during placebo and BPL1 treatment periods, Table S2. Basal demographic and physiologic characteristics of participants older than 4.5 years of age, Table S3. Absolute values of total body and abdominal fat mass at all visits, Table S4. Period effects on hyperphagia and physiologic outcomes, Table S5. Basal Childhood Behavior Check List (CBCL) scores of participants.
