# Supplementary material for: Effects of Bifidobacterium animalis Subsp. lactis (BPL1) Supplementation in Children and Adolescents with Prader–Willi Syndrome: A Randomized Crossover Trial
Source: Nutrients. 2020 Oct 13;12(10):3123. doi: 10.3390/nu12103123 (PMC7650793; doi:10.3390/nu12103123)
Supplement: Supplementary file 1 [file nutrients-12-03123-s001.zip › Amat-Bou et al Supplementary Information Nutrients.pdf]

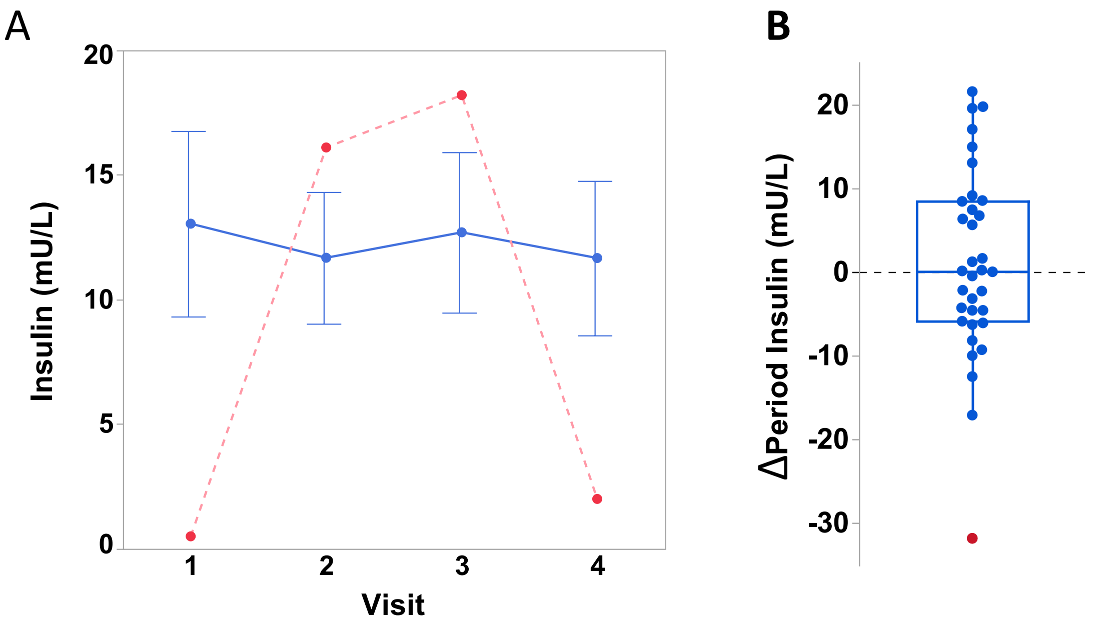

C

	**INSULIN (mU/L)**		**HOMA-IR**	
	**B (CI 95%)**	***P* value**	**B (CI 95%)**	***P* value**
**All subjects (n=34)**	-4.44 (-8.51, -0.38)	0.033	-1.07 (-2.04, -0.10)	0.031
**PW30 removed (n=33)**	-5.65 (-8.75, -2.55)	<0.001	-1.34 (-2.11, 0.58)	0.001

Supplemental Figure S1. Fasting insulin levels and change between periods.

A) Fasting insulin levels at all visits are shown as average (blue) and for subject PW30 (red). **B**) Difference of insulin levels between periods (Period 2-Period 1) was calculated; box plot shows median and interquartile range. **C**) Generalized linear regression was applied to assess treatment effects (sequence as independent variable) on insulin levels and HOMA-IR (dependent variables), adjusting for sex, age, genotype and basal fat mass; regression outcomes for "All subjects" are identical to values shown in Table 3 and are reproduced here for effect comparison to "PW30 removed". This patient is an 11-years-old female with basal BMI-SDS of -0.23; furthermore, she was born at 28 weeks of gestational age, being considered very preterm.

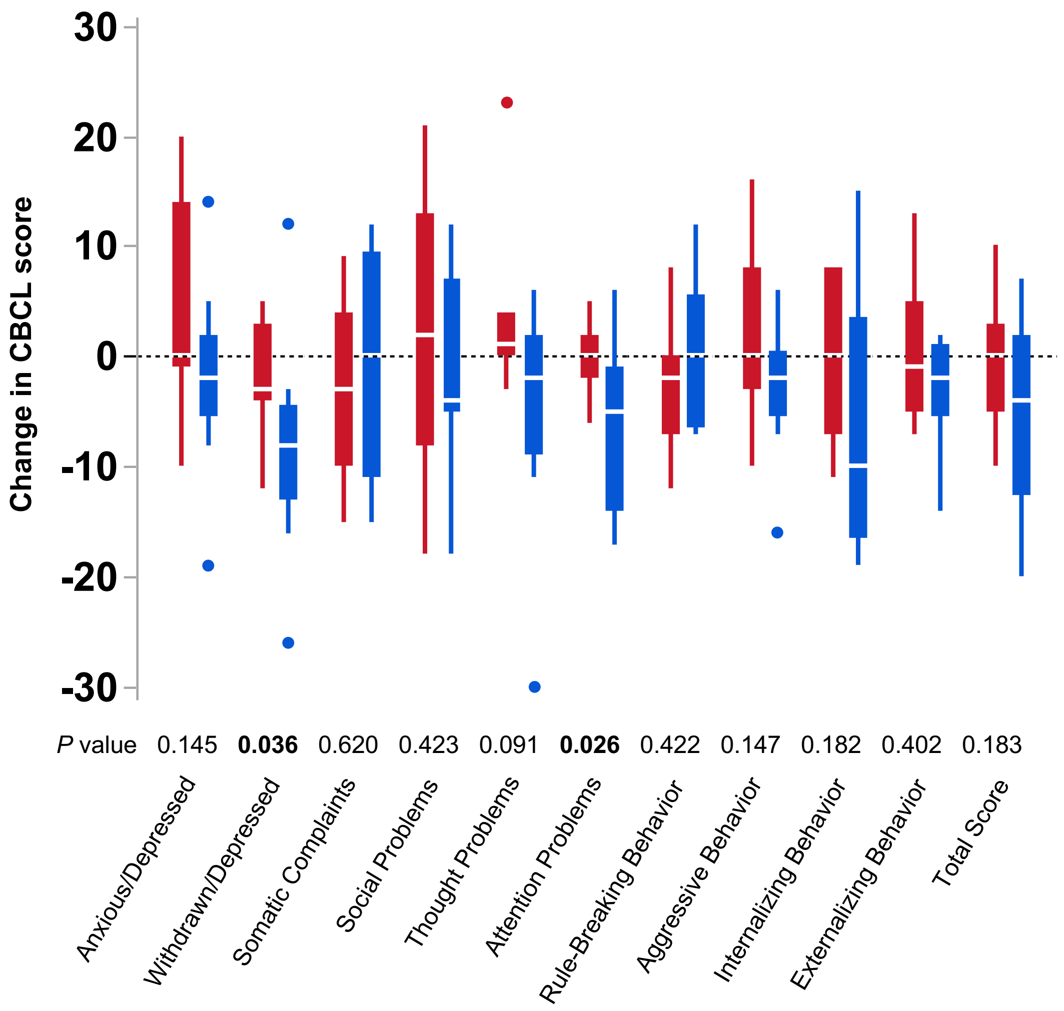

Supplemental Figure S2. Treatment effect on behavior by genotype.

Differences between BPL1 and placebo periods in CBCL scores (P_{BPL1}-$P_{Probiotic}$) for the indicated behavioral aspects are shown for subjects with deletion (n=11, red) or maternal disomy (n=9, blue). A change of 0 indicates no difference between BPL1 and placebo, and negative values indicate improvements with BPL1 versus placebo. Box plot shows median and interquartile range, with outliers (values above or below median ± 1.5 interquartile range are shown as red or blue circle. Differences between genotypes was assessed with the Wilcoxon test for each subscale.

Supplemental Table S1. Fold change of bacterial species during placebo and BPL1 treatment periods.

Supplemental table provided as a Microsoft Excel file.

Supplemental Table S2. Basal demographic and physiologic characteristics of participants older than 4.5 years of age.

	All (n=28)	AB (n=14)	BA (n=14)	*P* value
Sex (female)	17 (61)	10 (71)	7 (50)	0.243
Genotype (deletions)	15 (54)	9 (64)	6 (43)	0.254
Growth hormone use	27 (96)	14 (100)	13 (93)	0.233
Metformin use	8 (29)	6 (43)	2 (14)	0.089
Age (years)	12.1 (4)	12.1 (4.3)	12.1 (3.9)	0.969
Weight (kg)	55 (24.9)	56.1 (22)	53.9 (28.4)	0.825
Height (cm)	147.8 (17.7)	148.9 (18.3)	146.7 (17.7)	0.741
BMI-SDS	1.57 (1.34)	1.75 (1.18)	1.39 (1.51)	0.478
Body fat mass (g)	24437 (15321)	24523 (12598)	24350 (18133)	0.977
Body fat mass (%)	43.5 (8.5)	43.6 (7.7)	43.4 (9.5)	0.950
Abdominal fat mass (g)	1767 (1422)	1764 (1181)	1770 (1675)	0.992
Abdominal fat mass (%)	6.5 (1.7)	6.5 (1.9)	6.5 (1.5)	0.928
HQ-CT (Score)	6.4 (6)	6.7 (6.7)	6.1 (5.4)	0.806
Daily energy intake (kcal)	1585 (361)	1581 (431)	1588 (292)	0.962
Systolic pressure (mmHg)	111.6 (10.9)	111.9 (11.2)	111.4 (11)	0.906
Diastolic pressure (mmHg)	71.8 (8.5)	71.1 (8.7)	72.6 (8.6)	0.649
Triglycerides (mg/dl)	72 (26)	69 (23)	76 (28)	0.468
Total cholesterol (mg/dl)	170 (38)	177 (41)	162 (34)	0.306
LDL-cholesterol (mg/dl)	102 (32)	107 (34)	96 (31)	0.391
HDL-cholesterol (mg/dl)	57 (14)	57 (13)	57 (15)	0.883
Glucose (mg/dl)	87 (9)	84 (8)	90 (10)	0.077
HbA1c (%)	5.3 (0.2)	5.2 (0.1)	5.3 (0.3)	0.686
Insulin (mU/L)	13 (9.6)	12.2 (9.6)	13.9 (9.9)	0.640
HOMA-IR	2.91 (2.27)	2.63 (2.19)	3.19 (2.4)	0.527

Data are shown as mean and standard deviation (SD) for continuous variables and n and percentage (%) for categorical variables. Group differences were assessed with Student's t-test (continuous variables) or Chi-square test (categorical variables). HQ-CT, hyperphagia questionnaire for clinical trials; BMI, body mass index; BMI-SDS, body mass index standard deviation score; LDL, low-density lipoprotein; HDL, high-density lipoprotein; HOMA-IR, homeostatic model assessment of insulin resistance.

Supplemental Table S3. Absolute values of total body and abdominal fat mass at all visits.

	Sequence AB				Sequence BA			
	Placebo		Probiotic		Probiotic		Placebo	
	Visit 1	Visit 2	Visit 3	Visit 4	Visit 1	Visit 2	Visit 3	Visit 4
**Adiposity**								
**All subjects (n=35)**								
Body fat (g)	20037 (14017)	20111 (13734)	21104 (13978)	21162 (13883)	20743 (18213)	20901 (18654)	21711 (19684)	22335 (19321)
Body fat (%)	40.5 (9.7)	40.0 (9.4)	40.7 (9.6)	39.5 (9.3)	41.1 (10.0)	40.8 (10)	41.2 (10.1)	40.7 (9.4)
Abdominal fat (g)	1408 (1240)	1390 (1144)	1488 (1196)	1460 (1177)	1491 (1632)	1443 (1606)	1531 (1690)	1600 (1758)
**Subjects > 4.5 years-old (n=28)**								
Body fat (g)	24523 (12598)	24583 (12203)	25734 (12270)	25761 (12187)	24350 (18133)	24371 (18834)	25267 (19985)	25756 (19690)
Body fat (%)	43.6 (7.7)	43.1 (7.2)	44.0 (7.0)	42.4 (7.3)	43.4 (9.5)	42.3 (10.3)	42.6 (10.6)	41.6 (10.1)
Abdominal fat (g)	1764 (1181)	1738 (1060)	1852 (1105)	1813 (1097)	1770 (1675)	1698 (1667)	1792 (1760)	1863 (1838)
Abdominal fat (%)	6.5 (1.9)	6.6 (1.6)	6.8 (1.4)	6.6 (1.5)	6.5 (1.5)	6.2 (1.4)	6.3 (1.4)	6.4 (1.5)
**Insulin sensitivity**								
Insulin (mU/L)	9.9 (9.5)	10.3 (7.9)	11.6 (10.1)	9.6 (8.3)	12.7 (9.9)	9.9 (6.3)	10.0 (6.7)	11.9 (7.1)
HOMA-IR	2.14 (2.15)	2.19 (1.70)	2.60 (2.49)	2.08 (1.92)	2.89 (2.38)	2.19 (1.46)	2.19 (1.54)	2.63 (1.64)

Data are shown as mean (SD) at each visit from all subjects (n=35) or subjects older than 4.5 years of age (n=28). HOMA-IR, homeostatic model assessment of insulin resistance

Supplemental Table S4. Period effects on hyperphagia and physiologic outcomes.

	Period effect	
	**B (95% CI)**	***P* value**
Body fat mass (g)	160 (-531, 852)	0.639
Body fat mass (%)	-0.51 (-1.37, 0.34)	0.232
Abdominal fat mass (g)	43 (-33, 120)	0.258
Abdominal fat mass (%)	0.02 (-0.30, 0.34)	0.877
HQ-CT (Score)	-1.22 (-3.25, 0.81)	0.227
Energy intake (kcal/day)	-95 (-230, 40)	0.161
Systolic pressure (mmHg)	-2.00 (-7.96, 3.97)	0.495
Diastolic pressure (mmHg)	-0.07 (-6.34, 6.21)	0.983
Triglycerides (mg/dl)	-1.90 (-17.05, 13.24)	0.799
Total cholesterol (mg/dl)	0.28 (-10.61, 11.18)	0.958
LDL-cholesterol (mg/dl)	2.15 (-6.37, 10.67)	0.610
HDL-cholesterol (mg/dl)	-0.76 (-5.13, 3.61)	0.725
Glucose (mg/dl)	-1.37 (-6.64, 3.90)	0.600
HbA1c (%)	0.07 (-0.01, 0.14)	0.075
Insulin (mU/L)	1.16 (-2.78, 5.10)	0.552
HOMA-IR	0.24 (-0.7, 1.17)	0.612

Mutivariate generalized linear regression was applied to estimate period effects (n=35). Model was adjusted for sex, age, genotype, and basal % body fat mass. HQ-CT, hyperphagia questionnaire for clinical trials; LDL, low-density lipoprotein; HDL, high-density lipoprotein; HOMA-IR, homeostatic model assessment of insulin resistance; B, effect size; CI, confidence interval. A $P < 0.05$ was considered significant.

Supplemental Table S5. Basal CBCL scores of participants.

	**All subjects (n=20)**	**Deletions (n=11)**	**Disomies (n=9)**	***P* value**
Anxious/depressed	59.0 (8.7)	59.9 (10.1)	57.9 (6.9)	0.604
Withdrawn/depressed	61.5 (7.6)	62.1 (8.9)	60.7 (6.1)	0.679
Somatic complaints	61.4 (5.6)	62.4 (3.7)	60.2 (7.4)	0.444
Social problems	68.2 (9.4)	70.2 (9.8)	65.7 (8.8)	0.293
Thought problems	66.0 (9.0)	68.0 (9.1)	63.4 (8.7)	0.268
Attention problems	61.1 (5.2)	61.1 (5.4)	61.0 (5.2)	0.970
Rule-breaking behavior	58.7 (6.2)	60.9 (6.0)	55.9 (5.5)	0.068
Aggressive behavior	63.6 (9.0)	62.9 (8.4)	64.3 (10.1)	0.740
Internalizing	61.7 (7.2)	62.3 (7.8)	60.9 (6.9)	0.678
Externalizing	61.5 (8.0)	62.4 (7.1)	60.3 (9.3)	0.597
Total score	65.1 (7.1)	66.5 (6.8)	63.4 (7.5)	0.365

Data for the CBCL subscales as well as internalizing, externalizing, and total scores are shown as mean and standard deviation (SD). Group differences were assessed with Student's *t*-test. CBCL, Childhood Behavior Checklist.
